# Impact of Exercise on Vascular Function in Middle-Aged and Older Adults: A Scoping Review

**DOI:** 10.3390/sports10120208

**Published:** 2022-12-14

**Authors:** Antonio Bovolini, Ana Raquel Costa-Brito, Faber Martins, Guilherme Eustáquio Furtado, Gonçalo V. Mendonça, Carolina Vila-Chã

**Affiliations:** 1 Laboratory for the Evaluation of Sports Performance, Physical Exercise, and Health (LABMOV), Polytechnic of Guarda, 6300-559 Guarda, Portugal; 2 Research Center in Sports Sciences, Health Sciences, and Human Development (CIDESD), 5001-801 Vila Real, Portugal; 3 Neuromuscular Research Laboratory, Faculty of Human Motricity, University of Lisbon, Cruz Quebrada-Dafundo, 1495-751 Lisbon, Portugal; 4 Interdisciplinary Centre for the Study of Human Performance (CIPER), Faculty of Human Motricity, University of Lisbon, Cruz Quebrada-Dafundo, 1495-751 Lisbon, Portugal

**Keywords:** exercise training, macrovascular function, microvascular function, vascular ageing

## Abstract

There is a substantial literature gap related to the vascular response to different types of exercise training in middle-aged and older populations. Thus, this scoping review aimed to examine the outcomes of controlled trials testing the long-term effects of exercise interventions on vascular function-related outcomes in middle-aged and older populations. The literature search was conducted following PRISMA guidelines. Data sources: five databases were used (EBSCO, MEDLINE, Web of Science, Science Direct, and Google Scholar). Eligibility criteria: controlled trials, published in the last 10 years, in English, containing well-described exercise interventions, reporting vascular quantitative effects of exercise in middle-aged and older people. A total of 62 publications were included. The studies included distinct types and intensities of exercise and were heterogeneous in volume and frequency. The assessed vascular outcomes also presented considerable variability. Overall, most studies reported positive effects of exercise on vascular function outcomes, regardless of exercise characteristics. Different exercise interventions can be applied to improve vascular function in middle-aged and older adults. Studies on combined and stretching exercises reported encouraging results in improving vascular function. Stretching exercises rise as an effective alternative in promoting vascular function among older adults, while combined exercise delivered promising vascular benefits in both populations.

## 1. Introduction

Several ageing-related cardiovascular diseases (CVDs) are triggered by changes in the arterial phenotype [1,2], such as the stiffening of large elastic arteries and endothelial dysfunction, which are independent predictors of forthcoming CVDs [3]. Given the high prevalence of ageing-related vascular disorders in males and females [4,5], promoting vascular health and preventing the development of CVDs in middle-aged (40–60 years) and older adults (60 years or older) is critical for public health.

Based on recent reports from the American College of Sports Medicine, there is a growing demand for health-oriented physical exercise for specific populations in Europe and the world, including among middle-aged and older adults [6,7]. The latest World Health Organization guidelines highlight and reaffirm the beneficial role of physical activity in the general health of older adults [8]. Indeed, the role of exercise in reducing risk factors for CVD and preventing viral infection diseases (in particular by SARS-CoV-2) in middle-aged and old adults is well-established [9,10,11,12]. However, the positive impact of exercise is not only circumscribed to changes in CVD risk factors. Current evidence indicates a direct impact of exercise on arterial function (e.g., anti-atherogenic and anti-oxidative stress effects), mainly due to repetitive exposure to hemodynamic stimuli, such as shear stress and transmural pressure [13,14,15,16]. In this sense, exercise represents a promising approach to preventing/managing vascular dysfunction, particularly in middle-aged and older adults [17]. Exercise is a highly versatile non-pharmacological tool with few contraindications and minimal side effects [18]. Yet, the available literature on exercise and vascular function in older populations is relatively scarce. Most of the accumulated evidence is based on exposure to endurance exercise [19]. Nonetheless, few studies directly compared the same endurance exercise mode performed at different exercise intensities [20,21]. On the other hand, the effects of resistance and stretching training on vascular function remain poorly explored.

Given vascular senescence is typically accompanied by functional (physical and cognitive) limitations [22], investigating the potential benefits of diverse types of exercise in this population is critical. Despite the evidence indicating that exercise training represents an effective strategy to promote vascular health [13,23], there are inconsistent data on the effects of chronic exercise on the vascular function of middle-aged and older adults. In addition, the magnitude of structural arterial adaptation (e.g., measured through the flow-mediated dilatation) to distinct characteristics of exercise (e.g., intensity, volume), as well as exercise-induced changes in different vascular regions (e.g., central vs. peripheral), have been poorly characterized and investigated. Therefore, it is relevant to identify and systematize the primary benefits of exercise training on vascular function and characterize the training regimens to allow fine-tuning the exercise prescription based on safety and effectiveness.

This review aims to provide a comprehensive overview of controlled trials testing the long-term impact of different exercise modes on vascular function. Therefore, we presented a characterization of each exercise intervention and its impact on vascular function outcomes. Additionally, a characterization of the populations enrolled in the analysed studies is provided to improve clarity on the exercise effects.

## 2. Materials and Methods

### 2.1. Protocol and Registration

With a scoping review, we aimed to search for broader inclusion and search criteria to: (i) map the available evidence; (ii) methodologically characterize current research; (iii) identify the evidence’s important features; and (iv) identify current knowledge gaps. Likewise, it serves as a precursor to future and more accurate and valuable systematic reviews and meta-analyses on exercise and vascular function [24]. This review followed the Preferred Reporting Items for Systematic Reviews and Meta-Analyses extension for scoping reviews (PRISMA-ScR) guidelines [25] and the five-stage framework outlined in Arksey and O’Malley [26]. The approached research question was the following: “what is known about the long-term effects of exercise training on vascular function in middle-aged and older adults?” ‘Exercise’ is the planned, structured, and repetitive action of physical activity aimed at maintaining or improving health- or skill-related components of physical fitness [27,28]. ‘Vascular function’ refers to the conduit (blood distribution) and cushioning function (blood-flow maintenance) of the vascular system in delivery (gases/nutrients) as well as in arterial volumetric and pressure control (vasoconstriction/dilation) to maintain cellular homeostasis [29]. The review protocol from this study has not been registered/published.

### 2.2. Eligibility Criteria

The following criteria were applied to define the studies’ eligibility: published from January 2011 to November 2021 (to identify the most up-to-date relevant studies); in English; methodologically designed as randomized controlled trials (RCTs) or non-RCTs; containing well-described exercise interventions in the study methodology; and report quantitative effects of, at least, one vascular function outcome. Only middle-aged/older adults’ studies (mean age of 45 years or older—no upper limit) were included. Exclusion criteria: studies published in languages other than English, acute effects, literature reviews, and studies in which the exercise protocol was poorly or not described. Authors were contacted for missing information before study exclusion.

### 2.3. Search Strategy

The search strategy followed the eligibility criteria and the guidelines for evidence selection according to Tricco et al. and Arksey et al. [25,26]. Searches were conducted in November 2021 in the following databases: Elton Bryson Stephens Company (EBSCO), MEDLINE (PubMed), Web of Science, Science Direct, and Google Scholar. A preliminary search based on the study titles was performed and, if relevant, abstract, and full-text analysis.

The following terms were applied on the databases search engines: exercise OR physical exercise OR physical training OR (physical) exercise training OR (physical) exercise intervention OR (physical) exercise program followed by (AND) ‘vascular function’ term. The selected advanced search options included: controlled trials, publication range date, language, age group, and NO literature reviews. Reference lists of the included papers were checked for eligibility and additional papers were included if meeting the inclusion criteria.

### 2.4. Selection of Sources of Evidence

Title and abstract screening were carried out by three authors (AB, FM, and CVC). Full texts were independently reviewed by four authors (AB, FB, ARB, and CVC). There were no conflicts of analysis.

### 2.5. Data charting Process

Data charting followed the Arksey et al. Guidelines [26] and was undertaken by a single author (AB) using an extraction model specifically designed. Data extraction was done jointly by two authors (AB and FM) and individually checked by another three authors (CVC, GF, and ARB) following the review goals previously described. Data were obtained exclusively from the selected articles. Two authors (ARB and CVC) reviewed each study for vascular function indicators and exercise intervention.

### 2.6. Data Items

The following data were extracted: target population characterization (sample size, sex, mean age, mean body mass, body mass index, polypharmacy, and health status), exercise regimens (type of exercise, intensity, session duration, frequency, and intervention duration), and estimated vascular function indicators (described below). Since vascular responses are strongly linked to the specificities of exercise training, we focused on the intensity, duration, and type of exercise. The intensity of the exercise interventions was categorized as low-, low-to-moderated-, moderated, moderate-to-high-, or high-intensity [30].

The analyzed vascular outcomes were divided into two categories: (i) macrovascular (pulse wave velocity (PWV); augmentation index (ALX); flow-mediated dilatation (FMD); glyceryl trinitrate-induced dilation (GTN); cardio-ankle vascular index (CAVI); pulse stiffening ratio (PSR); intima-media thickness (IMT)); and (ii) microvascular (acetylcholine-mediated vasodilation (ACh); sodium nitroprusside endothelium-independent relaxant (SNP); reactive hyperaemia/index (RH/RHI)).

## 3. Results

### 3.1. Selection of Sources of Evidence

**Figure 1:**
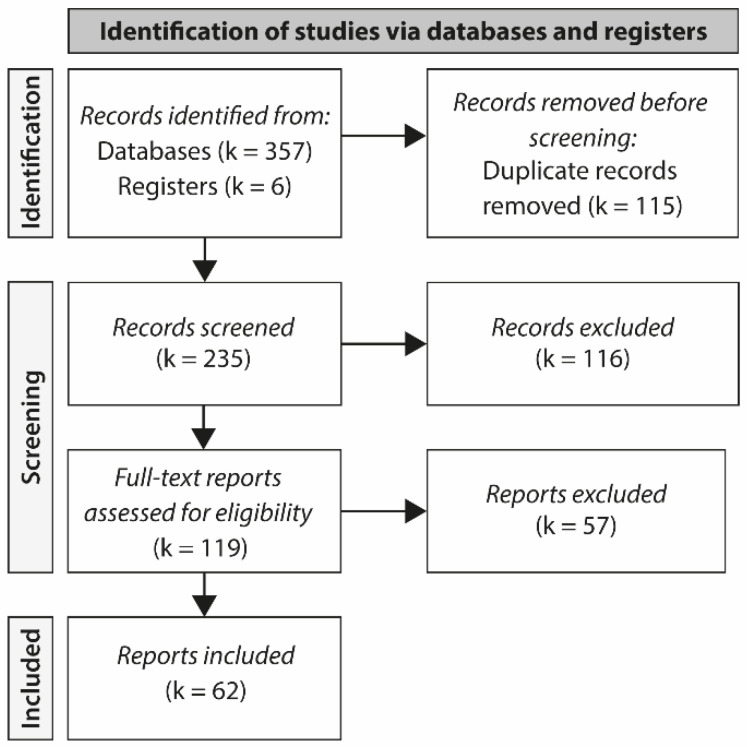
PRISMA flow diagram showing reasons for study exclusions. PRISMA, Preferred Reporting Items for Systematic Reviews and Meta-Analyses.

A total of 357 studies were selected from the databases for eligibility appraisal. After assessing the titles and abstracts of all entries, 119 studies were selected for full-text evaluation. As shown in Figure 1, following the screening of the full text of these reports, 62 studies met the inclusion criteria and were comprised in this scoping review.

### 3.2. Characteristics of Included Evidence Sources

As depicted in Supplementary Table S1, this review included 16 non-randomized [31,32,33,34,35,36,37,38,39,40,41,42,43,44,45,46] and 46 randomized controlled trials [38,47,48,49,50,51,52,53,54,55,56,57,58,59,60,61,62,63,64,65,66,67,68,69,70,71,72,73,74,75,76,77,78,79,80,81,82,83,84,85,86,87,88,89,90,91,92]. The characteristics regarding exercise interventions, target populations, and vascular outcomes, were ranked in three tables. Supplementary Table S1 describes the key features of exercise interventions. Supplementary Table S2 reports the major features of the target populations. Supplementary Table S3 lists the vascular outcomes quantified by the studies included in this review. Supplementary Tables S1–S3 are contained in the Supplementary Materials.

### 3.3. Target Population Features

The main characteristics of the population are described in Supplementary Table S2. Forty-seven included middle-aged adults [31,32,33,34,36,37,39,41,42,43,44,45,46,47,48,49,50,51,52,53,54,55,56,57,58,59,60,61,63,64,65,66,69,71,72,73,75,76,77,78,81,84,87,88,91,92,93], 15 studies included older adults [35,38,40,67,68,70,74,79,80,82,83,84,86,89,90], and four studies presented non-pooled groups of older and middle-aged adults [40,74,79,89]. Most of the studies (k = 43) comprised a mixed sample of males and females [32,35,36,37,38,40,43,44,45,46,49,50,51,53,54,55,57,58,59,60,62,63,65,66,68,70,71,72,73,74,76,77,78,79,80,81,82,83,84,87,89,90,92]. Fourteen studies included exclusively females [31,33,34,39,42,47,48,56,61,64,69,75,88,91], mainly in the middle-age, and five studies comprised only male participants [41,52,67,85,86]. In most studies (k = 32), participants presented a pre-obesity nutritional status (body mass index, BMI, greater than or equal to 25 to 29.9 kg/m^2^) [32,33,35,36,40,43,46,48,50,53,54,55,56,57,58,59,62,63,64,67,71,72,74,76,79,80,81,82,85,86,90,92]. In 15 studies the population had normal body mass index (BMI greater than or equal to 18.5 to 24.9 kg/m^2^) [31,39,40,41,42,43,45,49,55,69,74,77,84,85,94], 13 studies included participants with class 1 obesity (BMI between 30 to 34.9 kg/m^2^) [34,51,52,60,66,68,75,76,78,80,87,89,91]. Seven studies did not report BMI [37,38,47,61,65,73,88].

Regarding health status of the participants, 41 studies included persons with some disease (e.g., type 2 diabetes, peripheral artery disease) [32,33,34,35,37,38,40,46,48,49,50,51,53,54,55,56,57,58,59,60,62,63,64,66,67,68,69,70,71,72,75,76,78,79,80,82,84,85,87,89,92], 16 enrolled healthy individuals [31,41,42,43,45,47,52,61,65,74,77,81,86,88,90,91], and five studies did not report the populations’ health status [36,39,44,73,83]. Among pathologies (Supplementary Table S2), the most prevalent were cardiovascular diseases, such as hypertension (k = 7), heart failure (k = 4), coronary artery disease (k = 4), peripheral artery disease (k = 4), heart transplant (k = 1), Chagas heart disease (k = 1), followed by metabolic diseases, including type 2 diabetes (k = 8), and metabolic syndrome (k = 4). Forty-six studies reported medication intake [32,33,34,35,36,37,38,40,43,44,46,48,49,50,51,52,53,54,55,57,58,59,60,62,63,64,66,67,68,70,71,72,73,75,77,78,79,80,82,83,84,85,87,89,90,92].

### 3.4. Vascular Function Outcomes

**Figure 2:**
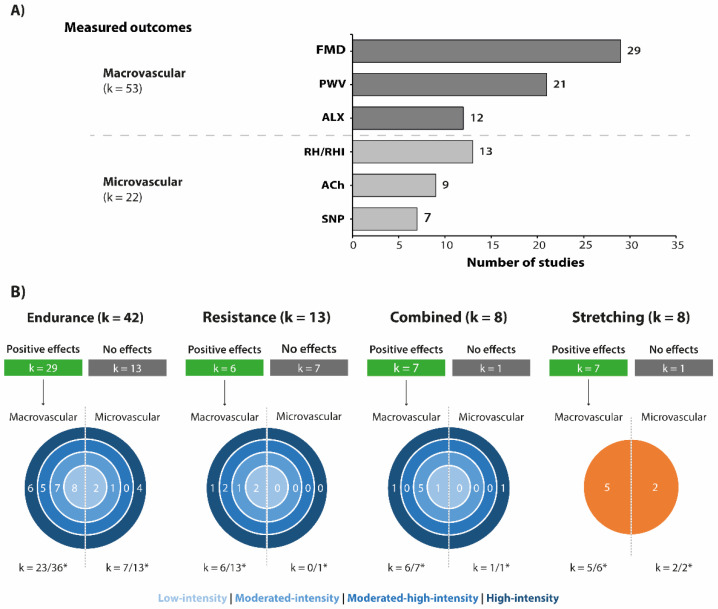
(**A**) Main vascular function outcomes according to exercise interventions. Graph bar presents the three most frequent macrovascular and microvascular measures used in the selected studies. (**B**) Diagram describing the number of studies that reported positive effects of exercise interventions on vascular function according to outcome category, exercise type, and exercise intensity. * Total number of studies that investigate at least one macro-/microvascular outcome. Acetylcholine-mediated vasodilation (ACh); Augmentation index (ALX); Flow-mediated dilatation (FMD); Pulse wave velocity (PWV); Reactive hyperemia/Reactive hyperemia index (RH/RHI); Sodium nitroprusside-induced endothelium-independent relaxant (SNP).

Among the categories of vascular indicators, the macrovascular outcomes were the most frequently assessed (k = 53) (Figure 2A). The most evaluated macrovascular outcomes were FMD (k = 29), PWV (k = 21), and ALX (k = 12). Macrovascular function outcomes were evaluated in approximately 86% of endurance exercise protocols, 100% of resistance exercise protocols, 88% of combined exercises, and 75% of the stretching exercise protocols. The microvascular outcomes were the less studied (k = 22, Figure 2A). Within this category, nine studies analysed the endothelial-dependent vasodilation (induced by ACh) and seven studies the endothelial-independent vasodilation (induced by SNP). The RH/RHI was assessed in 13 studies.

### 3.5. Types of Exercise Interventions

**Figure 3:**
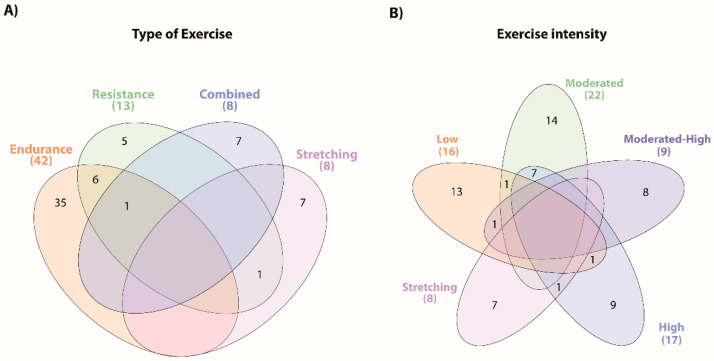
Summary of the main types of exercise training and intensities used in the interventions. The Venn diagrams present the overlap of the distinct types of exercise (**A**) and the different exercise intensities (**B**) described in the exercise interventions of the included studies.

Regarding the type of exercise, 54 studies used a single exercise type, with most studies focusing on endurance training (35 studies) [31,32,33,37,39,42,43,44,45,46,47,50,51,53,54,55,57,58,59,61,64,65,66,71,72,73,75,77,79,80,81,83,84,87,92] (Supplementary Table S1). Among the remaining studies, seven explored the impact of combined training [34,49,52,60,62,78,85], 7 on stretching exercises [38,41,48,68,70,88,91], and five focused on resistance training [35,36,56,67,69]. Regarding studies that compared different training approaches six studies compared endurance and resistance [40,63,74,76,82,86], one resistance and stretching [90], and one study compared endurance, resistance, and combined (endurance and resistance) interventions [89]. Figure 3 displays the diagrams of the distribution and combination of the interventions according to the exercise type and intensity.

Fifty-four studies presented just one type of exercise intensity: 13 studies on low-intensity exercises [31,34,39,42,45,47,50,56,66,69,80,81,84], 14 on moderated-intensity exercises [35,36,43,44,46,51,52,57,59,62,74,85,86,92], eight on moderated-high-intensity exercises studies [33,60,63,65,67,72,76,87], nine on high-intensity exercises [37,40,49,53,61,64,71,75,77], and seven studies with subjective intensity scales used for stretching exercises (predominantly RPE Scale, Supplementary Table S1) [38,41,48,68,70,88,91]. Eight studies involved exercise protocols with two different intensities: moderated- and low-intensity exercises [79], moderate- and high-intensity exercises [55,58,73,78,82,89], and low- and high-intensity exercises [54].

The training protocols also included different exercise modalities. Among the endurance interventions, cycling was the most frequent modality (k = 31), followed by walking/running (land, aquatic, or treadmill; k = 23). Less studied were the following modalities: elliptical (k = 4), rowing (k = 4), step climbing (k = 2), floorball (k = 2), swimming (k = 1), and football (k = 1) also made up the endurance exercise protocols (Supplementary Table S1). The resistance-type modalities were essentially centred on strength training with machines and/or free weights (k = 20).

**Figure 4:**
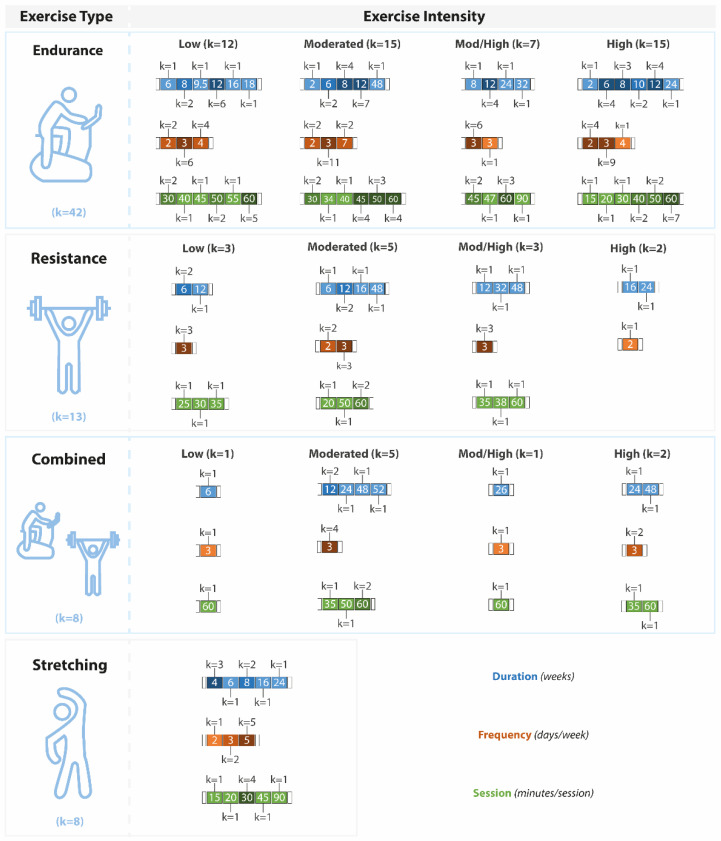
Characteristics of the exercise interventions (total duration in blue (weeks), frequency in brown (days per week), and the time of each session in green (minutes)). Exercise interventions are presented according to the exercise type and exercise intensity. Mod/High: moderate-high.

Most of the endurance interventions had a duration of 12 weeks, a frequency of three times per week, and 60 min per session (Figure 4). Regarding resistance training, there was considerable heterogeneity. In these studies, the overall duration of the intervention ranged from 6 to 48 weeks, and the duration of each training session varied between 20 and 60 min, two to three times per week. The duration of the combined exercise interventions ranged between 6 to 52 weeks, with a frequency of 3 days per week and a duration of 60 min per session. Similarly, the stretching interventions also shared a wide difference in training protocol characteristics. Overall, the exercise interventions lasted predominantly 4 to 8 weeks, with five training sessions per week of 30 min each.

Among the exercise types, about 87.5% of stretching, 87.5% of combined, 69% of endurance and 46% of interventions with resistance exercises reported at least one positive effect on vascular function outcomes (Figure 2B). Only one study explored the retention of vascular adaptations to exercise after a detraining period of 5 months [55].

## 4. Discussion

This scoping review examined the impact of different exercise interventions on the vascular function of middle-aged and older adults. Specifically, we described and systematized the characteristics of the exercise interventions and the specificities of the target populations exposed to the exercise interventions. Finally, we categorized and reported the impact of different exercise regimens on macro- and microvascular function outcomes.

### 4.1. General Characteristics of the Population

Ageing-related arterial maladaptation is a major risk factor for CVDs [95]. Therefore, understanding the exercise contributions to the age groups most vulnerable to ageing-related vascular changes is essential. Regular exercise appears to be the most effective non-pharmacological prophylactic/therapeutic strategy against ageing effects on vascular health regardless of sex [17,96]. However, the current literature presents a considerable gap in investigating the vascular response of middle-aged and older adults to exercise. Considering studies in populations over 75 years old, classified as old-old (75–94 years) and oldest-old (over 95 years) [97], our systematic review results revealed a lack of studies in these age groups. None of the studies included individuals over 72 years of age. This might be a consequence of the clinical characteristics usually associated with the ageing process, such as physical/cognitive decline, and frailty [22,98,99]. Such characteristics demand specific exercise interventions to meet the specific needs of these populations [22,98,99]. For instance, endurance and resistance exercises require functional and motor skills that are frequently incompatible with the physical-functional status of older individuals [98,99]. In this sense, less motor complex, and effort-demanding exercise interventions, such as stretching exercise, have emerged as an alternative to promote vascular health in middle-aged and older adults.

Most of the included studies investigated the vascular impact of exercise in middle-aged adults in mixed populations of males and females. However, rising evidence reveals that the progress of vascular ageing in females may follow a different chronology than in males, likely due to the role of sex hormones in the modulation of vascular (dys)function [100,101,102]. Yet, only middle-aged studies were composed of women.

Overall, the study population was characterized mainly by middle-aged adults, pre-obese, with some clinical condition (predominantly cardio-metabolic diseases), and consumers of some type of medication (little or nothing described in the included studies). In addition to the lack of studies dedicated to the older population, our results emphasize the lack of studies controlling confounding factors such as medication intake, menopause, previous levels of physical activity, and the control of exercise intensity.

### 4.2. Endurance Training

Studies with endurance exercise interventions showed the highest number of vascular benefits (29 studies). Among them, the benefits of aerobic training on vascular function were mostly in macrovascular outcomes. Endurance training was particularly effective in decreasing PWV (seven studies) and increasing FMD (20 studies). FMD is a well-established measure to assess the future risk of cardiovascular disease, being suggested as an independent predictor of cardiovascular events in different populations [103,104,105]. Likewise, PWV is the gold-standard method for arterial stiffness evaluation and a predictor marker of cardiovascular events in several clinical conditions such as heart failure, hypertension, and pulmonary hypertension [106]. Therefore, the modulation of these vascular health indicators represents a reduction in the risk of cardiovascular adverse events and diseases. Endurance interventions reporting vascular function benefits applied low- (eight studies) [31,39,50,66,80,81,83,84], moderated- (seven studies) [43,46,58,59,74,82,92], high- (six studies) [53,58,75,77,82,89], and the moderated-high- (five studies) [33,63,65,83,87] intensity of exercise. Endurance training also showed benefits on other vascular markers such as IMT (one study) [45]. However, this type of exercise was not effective in modulating other markers of the macrovascular function such as ABI, ALX, and CAVI at all exercise intensities, no studies reported any benefit of endurance training on these markers [39,44,55,71,72,75,83,84,86,92]. These markers are predictors of heart and vascular diseases commonly linked to ageing, such as arterial stiffness and atherosclerotic and coronary heart disease [107,108].

The benefits of endurance training on microvascular function were less investigated and the least reported by the studies (seven in 29 studies) [42,46,47,54,61,64,79]. Positive effects of endurance training on SNP (three studies) [46,47,64], ACh (five studies) [37,42,47,61,64,79], and RH (one study) [54] were reported. Comparing exercise intensities, a higher number of high-intensity endurance exercise studies reported benefits in microvascular function outcomes (five studies) [37,40,54,61,64]. Nevertheless, microvascular benefits were also reported in studies with moderated- (one study) [46], and low-intensity exercises (two studies) [42,47].

The vascular benefits of endurance training have been usually associated with relative intensity [109]. It has been suggested that high exercise intensities can induce greater shear stresses and more prominent vascular-related adaptations [110]. However, according to ACSM, high-intensity exercise may be particularly provocative for triggering negative cardiovascular events [111]. A substantial number of studies with low-intensity endurance exercise reported positive effects on at least one indicator of macrovascular function, suggesting such intensity is also effective in improving vascular function.

Overall, this review indicates that endurance exercise is effective to promote macrovascular function improvements, most notably in FMD, regardless of exercise intensity. Although a small number of studies had investigated microvascular (compared to macrovascular) benefits, endurance exercise was less effective in improving microvascular function. However, benefits in SNP, ACh, and RH were particularly reported from high-intensity endurance exercise interventions.

### 4.3. Resistance Training

Resistance training is a widely recommended non-pharmacological tool for preventing sarcopenia, osteoporosis, lifestyle-related diseases (e.g., diabetes), and maintaining and/or improving overall physical condition in middle-aged and older adults [112]. Resistance training is also known to reduce CVDs risk factors [113] but the mechanisms by which it reduces CVDs risk are still unclear. Although the potential benefits of resistance training on vascular function is a consistent hypothesis in the literature [114], the current evidence is still controversial and limited.

Approximately half of the studies reported some or no vascular benefit from resistance training (six studies reported improvements and seven studies indicated no benefits). Only macrovascular benefits from resistance training were reported. Among macrovascular outcomes, resistance training was effective in modulating FMD (three studies) [36,76,89], PWV (two studies) [56,63], and ALX (one study) [69]. However, it was not possible to identify the most effective exercise intensity, since beneficial effects were reported by studies with four different exercise intensities, but with similar frequency (low- [56,69], moderated- [36], moderated-high- [63,76], and high-intensity [89]). Such results seem to indicate that rather than exercise intensity, the frequency of the resistance training seems to be relevant to improve vascular function. Indeed, high-intensity resistance training has been associated with an increase in arterial stiffness markers in both normotensive and hypertensive adults [115]. Since vascular function is closely linked with the sympathetic nervous system, it has been reported that resistance training might increase arterial stiffness due to its strong sympathetic vasoconstrictive effect on arterial walls. The resistance training-related factors that contribute (or do not) to promoting vascular function are complex and the vascular adaptation mechanisms underlying resistance training are still not fully understood [116]. It is speculated that the mechanical compression of the vessels followed by the release of blood flow during resistance training seems to respectively induce transient ischemia and subsequent hyperaemia increasing local shear stress [116]. More studies are needed to investigate the mechanisms of vascular response to resistance training and optimize the prescription of this type of exercise to promote vascular health. However, the present review suggests that resistance training seems effective in positively modulating macrovascular function as half of the studies reported some positive effects on markers of macrovascular function, mostly decreasing PWV and increasing FMD. Given that no studies reported microvascular benefits, resistance training does not seem efficient in promoting microvascular function at any exercise intensity.

### 4.4. Combined Exercise Training

Combined exercise interventions showed a significant number of vascular benefits. Seven [34,49,52,62,78,85,89] of eight studies reported some beneficial vascular effects from combined exercise intervention. Only one study of moderate-high intensity combined exercise did not report any effect on vascular function [60]. As observed in the interventions of other types of exercises, macrovascular function outcomes were the most frequently analysed, being reported in seven studies [34,52,60,78,85,89], while only one study [49] examined the microvascular function outcomes. Among the studies analysing the effects of combined exercise on macrovascular function, six reported positive effects, namely on PWV (three studies) [62,78,85], FMD (two studies) [52,89], IMT (one study) [78], and ALX (one study) [34]. Among the exercise intensities from the studies showing beneficial macrovascular effects are moderated-intensity (five studies) [52,62,78,85,89], followed by high- (two studies) [78], and low-intensity (one study) [34] exercise. In contrast, only one study [49] looked at the impact of combined exercise of high-intensity on microvascular function outcomes, which revealed a positive modulation of the SNP and RH indicators. SNP is a commonly used vasodilator in pharmaceutical therapies and research to assess vessel dilation capacity in response to nitric oxide [117]. Along with RH, it is a significant indicator of microvascular and endothelial function [117].

The present review suggests that the combination of endurance and strength exercises is the most effective exercise combination for promoting vascular health since all studies showing positive vascular effects of exercise utilised this combination of exercise. Indeed, current literature suggests that the combination of endurance-and resistance-type exercises is associated with the improvement, or at least stabilization, of arterial stiffness markers in older adults [118]. Combined exercise training (compared to other modalities such as strength training and under the most recent international guidelines) has been identified as the most effective modality in improving different cardiometabolic parameters in adults, namely in adult populations with overweight or obesity [119]. However, the order in which endurance and resistance exercises are performed in combined exercise interventions may alter the vascular impact of this type of exercise [120,121,122]. More significant vascular benefits were reported when endurance training was performed after resistance exercises in middle-aged adults. Such results suggest that the order of execution in the combination of aerobic and strength exercises should be taken into consideration when prescribing combined training. However, more studies dedicated to examining the impact of different types and intensities of combined exercises are urgently needed.

### 4.5. Stretching Training

Interventions with stretching exercises also showed positive results in vascular function outcomes, with seven of eight studies [41,48,68,70,88,90,91] describing some vascular benefits. The macrovascular function outcomes were the most reported among studies with stretching exercise interventions (six studies). Among them, five studies report some benefits [41,48,68,90,91] regardless of intervention duration, training frequency, and training session duration. There was no predominance among the macrovascular function markers that most benefited from stretching exercises, with positive effects being reported on PWV (two studies) [41,48], ALX (two studies) [90,91], CAVI (one study) [41], and FMD (one study) [68]. Among the perceived intensities of stretching exercises, a higher number of vascular benefits were reported by stretching interventions with “minimal discomfort” intensity (four studies) [41,48,68,70]. Only one study with “fairly light” perceived intensity of stretching exercise did not report any effects on vascular outcomes [38]. The mechanisms underlying the beneficial effects of stretching exercise on vascular function are unclear, evidence has suggested that fluctuations in the shear rate during repetitive administration of stretching exercises are effective stimuli in improving vascular function and blood flow control mechanisms [123]. Still, it is important to highlight that after stretching training cessation, the improvements related to the central mechanisms remain for approximately 6 weeks, while the gains related to the local mechanisms seem to have a more persistent duration [123].

Only two studies [70,88] investigated microvascular function outcomes and both reported beneficial effects of exercise. Both studies were particularly efficient in increasing RH-PAT at “minimal discomfort” [70] and “somewhat heavy” [88] intensities of stretching exercise. RH/RH-PAT is a well-established method for the non-invasive assessment of microvascular (peripheral) function and a well-accepted predictor of all-cause and cardiovascular morbidity and mortality [124]. Despite the low number of studies dedicated to assessing the impact of stretching exercises on microvascular function outcomes, the results are promising. Stretching exercise protocols have recently gained attention from researchers as an ascending exercise alternative to promote vascular health, especially among older adults [94,123,125]. This type of exercise is highly versatile and feasible to implement in community-dwelling middle-aged and older adults [126]. Additionally, above all, stretching exercise protocols do not require highly specialized spaces and significant financial investment.

Overall, stretching exercise interventions were effective in promoting macro- (modulating CAVI, FMD, PWV, and ALX) and microvascular function (increasing RH-PAT), particularly at low perceived exercise intensities classified as “minimal discomfort”.

## 5. Strengths and Limitations

This is the first study that summarizes the main characteristics of interventions with different types of exercise intended to improve vascular function in middle-aged and older adults. This review included five distinct databases of search, providing a wide review of the subject. However, this review presents some limitations. First, the number of potential publications not identified in the systematic review is unclear. Accidental omission of relevant articles could potentially bias the results. Second, the diverse health status of the participants might limit the identification of types and intensities of exercise most effective in promoting vascular health in these populations. Finally, the low number of studies exploring the microvascular function response to exercise interventions did not allow a fair analysis of the related evidence.

## 6. Conclusions and Future Perspectives

This review presents the evidence of the beneficial effects of distinct exercise interventions in promoting vascular health in middle-aged and older adults. Among the most significant findings, our results point out that studies on combined and stretching exercises reported encouraging results for vascular function in both populations. Additionally, our study particularly emphasises that stretching exercises emerge as an efficacious alternative in promoting vascular function among older adults. In contrast, combined exercises have promising vascular benefits in both middle-aged and older adults. Given the adaptive capacity of exercise technical features, this review also found outcomes on the safety and high applicability of exercise interventions in populations with different clinical characteristics. Studies on combined and stretching exercise interventions reported promising outcomes in improving vascular function. Findings from this review will be valuable in designing forthcoming exercise interventions attempting to promote vascular health in both populations. However, further studies are necessary to fill important gaps identified in the current literature, such as: (i) the low number of studies exploring the vascular response to exercise in older adults; (ii) the low number of studies analysing the exercise effects on microvascular function; (iii) the exercise impact on sexual dimorphism related to vascular ageing; (iv) the vascular response to execution order between aerobic and resistance exercises in combined-exercise interventions; (v) the lack of follow-up studies on vascular responses to exercise; (vi) determining the dose-response between exercise and vascular function; and (vii) establishing standardized exercise protocols with different types of exercises, expanding alternatives for populations with special needs.

## Data Availability

Not applicable.

## Supplementary Materials

The following supporting information can be downloaded at: https://www.mdpi.com/article/10.3390/sports10120208/s1. Table S1: Exercise interventions characteristics; Table S2: Population characteristics; and Table S3: Vascular outcomes. References [26,27,28,29,30,31,32,33,34,35,36,37,38,39,40,41,42,43,44,45,46,47,48,49,50,51,52,53,54,55,56,57,58,59,60,61,62,63,64,65,66,67,68,69,70,71,72,73,74,75,76,77,78,79,80,81,82,83,84,85,86,87,88,89,90,91,92] are cited in the Supplementary Materials.
