# Supplementary material for: Impact of Exercise on Vascular Function in Middle-Aged and Older Adults: A Scoping Review
Source: Sports (Basel). 2022 Dec 14;10(12):208. doi: 10.3390/sports10120208 (PMC9786288; doi:10.3390/sports10120208)
Supplement: Supplementary file 1 [file sports-10-00208-s001.zip › Table S1-Exercise interventions.pdf]

Table S1. Exercise interventions characteristics

Author, *year* *(Reference)*	***Study Design***	***Age group***	***Exercise Modalities***	***Intensity of Exercise***	***Intervention Duration*** *(weeks)*	***Training Frequency*** *(days/week)*	***Training Session Duration*** *(min/session)*	***Effects of exercise on vascular function***
**Type of exercise: Endurance**								
Afousi, *2018* *(**45**)*	RCT	Middle-age	Walking / running	High-intensity	12	3	50 – 60	The exercise intervention increased brachial artery FMD (p = 0.01) in type 2 diabetes patients. The improvements were more significant in high-intensity training group.
Benda, *2015* *(**27**)*	Non-RCT	Middle-age	Cycling	High-intensity	12	2	45 – 50	No effects on brachial and superficial femoral FMD.
Conraads, *2015* *(**85**)*	RCT	Middle-age	Cycling<br>Cycling	High-intensity<br>High-intensity	12	3	30 – 60<br>60	Increase of FMD and improvement of resting diastolic BP after training, regardless of intervention, in patients with coronary artery disease.
Dall, *2015* *(**87**)*	RCT	Middle-age	Cycling	High-intensity	12	3	30	The exercise intervention had any effect on RHI or ALX. Loss of intervention impact 5 months after exercise cessation.
da Silva, *2012* *(**86**)*	RCT	Middle-age	Walking / running	High -intensity	6	4	50	High-intensity training improved RH-induced dilation compared with low-intensity training ($p < 0.05$) in patients with metabolic syndrome and type 2 diabetes.
Gliemann, *2020* *(**48**)*	RCT	Middle-age	Floorball	High-intensity	10	2	40 – 60	HIIT increase vascular responsiveness to ACh ($p < 0.05$). No effects were observed on mean BP, blood flow, and LVC.
Gunnarsson, *2020* **(51)**	RCT	Middle-age	Floorball	High-intensity	10	2	40 – 60	HIT improved the vasodilator response mediated by infusion of SNP ($p < 0.05$) and Ach ($p < 0.05$).
Hellsten, *2012* *(**38**)*	Non-RCT	Middle-age	Cycling	High-intensity	8	3	60	No effects were observed on LVC with ACh infusion between the groups before or after training.

Keech**, 2020* ***(59)	RCT	Middle-age	Cycling	High-intensity	6	2	15	No effects were observed on fePWV and ALX.
Klonizakis**, 2014* ***(61)	RCT	Middle-age	Cycling	High-intensity	2	3	40	No effects were observed on GTN-mediated dilation, baFMD and RH.
Lee**, 2019* ***(63)	RCT	Middle-age	Cycling	High-intensity	8	3	40	Increase of brachial artery FMD ($p = 0.005$) after exercise intervention in breast cancer patients. No effect of exercise intervention on IMT.
Luk**, 2011* ***(66)	RCT	Middle-age	Treadmill, ergometer, rowing, steps, arm ergometer, dumbbell and weight training	High-intensity	8	3	60	Increase of brachial artery FMD ($p = 0.002$) after exercise intervention in patients with coronary artery disease. No effect of exercise on GTN.
Munch**, 2018* ***(41)	Non-RCT	Middle-age/ older	Cycling	High-intensity	6	3	60	Increase of ACh-induced vasodilation and in patients with heart failure ($p < 0.05$).
O'Brien**, 2020* ***(71)	RCT	Older	Cycling	High-intensity	6	3	40	HIIT increased baFMD (both p < 0.001), pop-FMD and L-FMC (both, p < 0.045). HIIT improved BA L-FMC (p < 0.001).
Vinet**, 2018* ***(79)	RCT	Middle-age/ older	NR	High-intensity	24	NR	NR	Improvement of large vessels vascular function (FMD) after exercise intervention in metabolic syndrome patients ($p < 0.001$).
Cornelissen**, 2014* ***(34)	Non-RCT	Middle-age	Cycling, running, arm ergometer, rowing and calisthenics	Moderated-high-intensity	12	3	90	Exercise intervention increased FMD (p < 0.0001).<br>No effects were observed on RHI.
Greenwood**, 2015* ***(50)	RCT	Middle-age	Treadmill + elliptical	Moderated-high-intensity	12	3	47	Endurance training improved PWV in kidney transplant recipients ($p < 0.001$).
Haynes**, 2021* ***(52)	RCT	Middle-age	Land walking / jogging	Moderated-high-intensity	24	3	60	Increase of brachial artery FMD after land walking intervention only ($p = 0.034$) in sedentary older adults. No effects on brachial
			Water walking / jogging	Moderated-high-intensity				

								dilation induced by GTN, regardless of training intervention.
***Kirkman**, 2019 (**60**)*	RCT	Middle-age	Cycling, walking/jogging, or elliptical	Moderated-high-intensity	12	3	45	No effects on ALX and carotid/femoral FMD after exercise intervention in non-dialysis chronic kidney disease patients. Exercise maintained conduit artery endothelial function (prevention of FMD and RH decline).
***Lekavich**, 2021 (**65**)*	RCT	Middle-age	Cycling, walking, stair climbing or elliptical	Moderated-high-intensity	32	3	60	Aerobic training had no effect on FMD.
***Park SY**, 2020 (72)*	RCT	Middle-age	Land gait	Moderated-high- intensity	12	4	60	Exercise intervention decreased leg and brachial-to-ankle arterial PWV in patients with peripheral artery disease ($p < 0.5$). None effect on ABI.
***Schreuder**, 2014 (77)*	RCT	Middle-age	Cycling	Moderated-high-intensity	8	3	45	Exercise intervention improved baFMD/GTN (both $p < 0.05$). No effects were observed on baFMD, baGTN, and feFMD.
***Afousi**, 2018 (**31**)*	RCT	Middle-age	Cycling	Moderated-intensity	12	3	60	Exercise interventions increased brachial artery FMD ($p = 0.01$) in type 2 diabetes patients. However, improvements were more significant in high-intensity training group.
***Benda**, 2015 (**27**)*	Non-RCT	Middle-age	Cycling	Moderated-intensity	12	2	30	No effects on brachial and superficial femoral FMD.
***Chacaroun**, 2020 (**83**)*	RCT	Middle-age	Cycling	Moderated-intensity	8	3	20 – 60	No effects were observed on RHI and PWV.
***Dall**, 2015 (**87**)*	RCT	Middle-age	Cycling	Moderated-intensity	12	3	45	The exercise intervention had any effect on RHI or ALX. Loss of intervention impact 5 months after exercise cessation.
***Gainey**, 2016 (**82**)*	RCT	Middle-age	Treadmill	Moderated-intensity	12	3	30	Increase of FMD after exercise intervention in patients with type 2 diabetes ($p < 0.05$). No effects on ABI.

Gelinas, *2017 (**44**)*	RCT	Middle-age	Upper and lower cycling ergometers	Moderated-intensity	8	3	35 – 45	No effect on FMD after exercise training.
Gholami, *2020 (**46**)*	RCT	Middle-age	Cycling	Moderated-intensity	12	3	30 – 45	Increase of FMD after exercise intervention in patients with type 2 diabetes and peripheral neuropathy ($p = 0.0001$).
Klonizakis, *2014 (**45**)*	RCT	Middle-age	Cycling	Moderated-intensity	2	3	40	No effects were observed on GTN-mediated dilation, baFMD and RH.
Kujawski, *2018 (**62**)*	RCT	Middle-age/ older	Callisthenic	Moderated-intensity	12	2	50	Callisthenic training improved aortic stiffness (PWV, $p < 0.05$) in healthy older adults.
Munch, *2018 (**68**)*	*RCT*	Middle-age/ older	Cycling	Moderated-intensity	6	3	45	No alterations in vascular function (FMD and leg blood flow during active leg movement) after training.
O'Brien, *2020 (**81**)*	RCT	Older	Cycling	Moderated-intensity	6	3	34	Moderate-intensity continuous training increased baFMD (both $p < 0.001$), pop- FMD and L-FMC (both, $p < 0.045$).
Pierce, *2016 (**31**)*	Non-RCT	Middle-age	Walking	Moderated-intensity	8	6 – 7	45 – 50	No effects were observed on aortic ALX and aortic PWV.
Pierce, *2011 (**30**)*	Non-RCT	Middle-age	Walking	Moderated-intensity	8	6 – 7	40 – 50	Exercise intervention increased baFMD ($p < 0.001$) in middle-aged men but not in postmenopausal women.
Schmidt, *2014 (**76**)*	RCT	Older	Football	Moderated-intensity	48	2 – 3	60	No effects of football and strength training on RHI and ALX.
van Zanten, *2019 (**33**)*	Non-RCT	Middle-age	Treadmills, cycling, hand and rowing ergometers	Moderated-intensity	12	3	60	Improvement of vascular function (FMD, GTN and SNP) after exercise intervention ($p < 0.05$).
Akazawa, *2012 (**26**)*	Non-RCT	Middle-age	Cycling + walking	Low-intensity	8	3	30 – 60	Increase of brachial artery FMD after exercise training ($p <0.05$) in postmenopausal women.
Alkatib, *2014 (**42**)*	RCT	Middle-age	Treadmill	Low-intensity	8	2	40	Improvement of cutaneous vascular conductance induced by SNP and ACh in the lower-limb and the upper-limb ($p < 0.05$) after exercise intervention in sedentary postmenopausal women.

Bouaziz, *2019* ***(75)***	RCT	Middle-age	Cycling	Low-intensity	9,5	2	30	Improvement of brachial artery FMD after exercise intervention (*p* < 0.05) in sedentary older adults. No effect of exercise training on carotid/radial and carotid/femoral FMD.
da Silva, *2012* ***(86)***	RCT	Middle-age	Walking / running	low -intensity	6	4	50	No effect on RH-induced dilation in patients with metabolic syndrome and type 2 diabetes.
Headley, *2014* ***(54)***	RCT	Middle-age	Cycling, walking/jogging, elliptical, rowing	Low-intensity	16	3	55	No effects were observed on aPWV.
Koshiba, *2019* ***(40)***	Non-RCT	Middle-age	Cycling	Low-intensity	12	2 – 3	50	Decrease of brachial-ankle PWV after exercise intervention (*p* < 0.001. *p* < 0.01 and *p* < 0.05, respectively) in middle-aged females. No effect on CAVI.
Novaković, *2019* ***(69)***	RCT	Older	Walking + cycling	Low-intensity	12 – 18	3	60	Exercise intervention increased FMD (p = 0.002) and decreased PWV (p = 0.013).
Nualnim, *2012* ***(70)***	RCT	Middle-age	Swimming	Low-intensity	12	3 – 4	12 – 45	Exercise intervention increased FMD (p < 0.05). No effects were observed on femoral-ankle PWV and carotid ALX.
Nyberg, *2016* ***(29)***	Non-RCT	Middle-age	Cycling	Low-intensity	12	3	60	Exercise training improved Ach-induced vasodilation in postmenopausal women (*p* < 0.05).
Park SY, *2020* ***(72)***	RCT	Middle-age	Aquatic gait	Low-intensity	12	4	60	Exercise intervention decreased leg and brachial-to-ankle arterial PWV in patients with peripheral artery disease (*p* < 0.5). None effect on ABI.
Park S-Y, *2019* ***(73)***	RCT	Older	Walking	Low-intensity	12	4	60	Exercise intervention decreased femoral-to-ankle PWV (p < 0.05). No effects were observed on ABI.
Tanahashi, *2017* ***(32)***	Non-RCT	Middle-age	Cycling or walking	Low-intensity	12	3	30	Exercise intervention improves conduit artery decreasing baIMT (*p < 0.05)*. No effects were observed on NID (at rest and 10 min after GTN).

**Type of exercise: Resistance**								
***Vinet**, 2018 (**79**)*	RCT	Middle-age/ older	NR	High-intensity	24	NR	NR	Improvement of large vessels vascular function (FMD) after exercise intervention in metabolic syndrome patients (*p* < 0.001).
***Williams**, 2013 (**80**)*	RCT	Older	Strength	High-intensity	16	2	NR	No effect on ALX.
***Greenwood**, 2015 (**50**)*	RCT	Middle-age	Strength	Moderated-high-intensity	12	3	47	Resistance training improved PWV in kidney transplant recipients (*p* < 0.001).
***Hildreth**, 2018 (**55**)*	RCT	Older	Strength	Moderated-high-intensity	48	2 – 3	20 – 35	No effects of exercise training on carotid IMD and brachial artery FMD in older men.
***Lekavich**, 2021 (**65**)*	RCT	Middle-age	Strength	Moderated-high-intensity	32	3	60	Resistance training increased FMD (*p* = 0.004).
***Grafe**, 2018 (**36**)*	Non-RCT	Older	Strength	Moderated-intensity	12	2 – 3	20	No effect on RH-induced FMD.
***Green**, 2014 (**37**)*	Non-RCT	Middle-age	Strength	Moderated-intensity	8 – 16	2	NR	Exercise intervention increased baFMD (p < 0.001).
***Kujawski**, 2018 (**62**)*	RCT	Middle-age/ older	Strength	Moderated-intensity	12	2	50	No effects of resistance training in PWV.
***O'Brien**, 2020 (**71**)*	RCT	Older	Strength	Moderated-intensity	6	3	40	No effect on baFMD, pop- FMD and L-FMC.
***Schmidt**, 2014 (**76**)*	RCT	Older	Strength	Moderated-intensity	48	2 – 3	60	No effects of strength training on RHI and ALX.
***Eigendorf**, 2019 (**43**)*	RCT	Middle-age	Strength	Low-intensity	6	3	20	Exercise intervention decreased PWV (p = 0.057).
***Jaime**, 2019 (**57**)*	RCT	Middle-age	Strength	Low-intensity	12	2 – 3	20 – 35	Whole-body exercise intervention decreased ALX and ALX 75% (both p < 0.05). No effects were observed on PWV.
***Munch**, 2018 (**68**)*	*RCT*	Middle-age/ older	Strength	Low-intensity	6	3	25	No alterations in vascular function (FMD and leg blood flow during active leg movement) after training.
**Type of exercise: Combined**								

Borges, *2021* ***(64)***	RCT	Middle-age	Treadmill + strength	High-intensity	24	3	60	Improvement of cutaneous vascular conductance induced by SNP and RH (p = 0.05) after exercise intervention in patients with Chagas heart disease. No effect on cutaneous vascular conductance induced by ACh.
Magalhães, *2019* ***(67)***	RCT	Middle-age	Cycling + strength	High-intensity	48	3	35	Exercise intervention decreased carotid artery IMT (p < 0.05) and improved peripheral arterial stiffness indices (carotid distal PWV and carotid radial PWV, p < 0.05) in patients with type 2 diabetes.
Gibbs, *2012* ***(47)***	RCT	Middle-age	Treadmill/ cycling + strength	Moderated-high-intensity	26	3	60	Exercise intervention had no effects on brachial artery FMD in patients with type 2 diabetes.
Chasland, *2021* ***(84)***	RCT	Middle-age	Strength + cycling	Moderated-intensity	12	2 – 3	50	Increase of brachial artery FMD (p = 0.033) after exercise intervention in men aged 50 to 70 years. No effect on GTN dilation.
Greenwood, *2015* ***(49)***	RCT	Middle-age	Cycling + strength	Moderated-intensity	52	3	45 – 60	Positive effect of exercise intervention on carotid/femoral PWV (p < 0.001) in patients with progressive chronic kidney disease.
Magalhães, *2019* ***(67)***	RCT	Middle-age	Cycling + strength	Moderated-intensity	48	3	35	Exercise intervention decreased carotid artery IMT (p < 0.05) in patients with type 2 diabetes.
Park W, *2020* ***(74)***	RCT	Older	Treadmill / bicycle + strength	Moderated-intensity	12	3	60	Reduction of baPWV in obese older men (p < 0.001).
Vinet, *2018* ***(79)***	RCT	Middle-age/ older	NR	Moderated-intensity	24	NR	NR	Improvement of large vessels vascular function (FMD) after exercise intervention in metabolic syndrome patients (p < 0.001).
Eleutério-Silva, *2013* ***(35)***	Non-RCT	Middle-age	Treadmill + strength	Low-intensity	6	3	60	Exercise intervention reduced ALX (p = 0.026).
**Type of exercise: Stretching**								

Boonpin, *2017* ***(53)***	RCT	Middle-age	Active - static	Minimal discomfort	6	5	30	Stretching training improved baPWV (P < 0.05).
Hotta, *2019* ***(56)***	RCT	Older	Passive (splint) - static	Minimal discomfort (any pain)	4	5	30	Stretching training increase FMD (*p = 0.005*). No effects were observed on NID (at rest and 10 min after NTG).
Kato, *2017* ***(58)***	RCT	Older	Active - static	Minimal discomfort (any pain)	4	3	20	Stretching training increased RH-PAT index *(p < .05)*
Kim, *2019* ***(39)***	Non-RCT	Older	Active + passive - static	RPE ≤ 11 "fairly light" (Borg scale)	8	3	90	No effects were observed on ABI and CAVI.
Nishiwaki, *2015* ***(28)***	Non-RCT	Middle-age	Active - static	Minimal discomfort (mild stretching)	4	5	30	Stretching training induced a reduction in arterial stiffness: reducing baPWV (*p < 0.05*) and CAVI (*p < 0.05*).
Shinno, *2017* ***(78)***	RCT	Middle-age	Active - static	"Somewhat heavy" to "Heavy" (RPE-Borg Scale)	12 – 24	5	15	Stretching training improved arterial stiffness increasing RH-PAT (*p < 0.01*). Stretching training effects were reversed by detraining.
Williams, *2013* ***(80)***	RCT	Older	Flexibility	NR	16	2	45	Flexibility training increased ALX (*p < 0.05*).
Wong, *2014* ***(81)***	RCT	Middle-age	Active + passive - static	Maximal exertion (RPE>18)	8	5	30	Stretching training increased aortic ALX (*p < 0.05*).

Ankle brachial index (**ABI**); Acetylcholine (**ACh**); Augmentation index (**ALX**); Brachial-ankle PWV (**baPWV**); Cardio-ankle vascular index (**CAVI**); Flow-mediated dilatation (**FMD**); Glyceryl trinitrate-response (**GTN**); High intensity training (**HIT**); High intensity interval training (**HIIT**); Intima-media thickness (**IMT**); Low flow-mediated constriction (**L-FMC**); Leg vascular conductance (**LVC**); Nitroglycerin-induced dilation (**NID**); Not reported (**NR**); Nitroglycerin (**NTG**); Pulse wave velocity (**PWV**); Randomized controlled trial (**RCT**); Reactive hyperemia (**RH**); Reactive hyperemia peripheral arterial tonometry index (**RH-PAT**); Rating of Perceived Exertion (**RPE**); Sodium nitroprusside (**SNP**).
