# Supplementary material for: Impact of Exercise on Vascular Function in Middle-Aged and Older Adults: A Scoping Review
Source: Sports (Basel). 2022 Dec 14;10(12):208. doi: 10.3390/sports10120208 (PMC9786288; doi:10.3390/sports10120208)
Supplement: Supplementary file 1 [file sports-10-00208-s001.zip › Table S2-Population characteristics.pdf]

Table S2. Population characteristics

***Author**, year (Reference)*	***Sex***	***Number of participants***	***Mean age***	***Mean body weight** (kg)*	***BMI** (kg/m²)*	***Nutritional status ****	***Medicine intake***	***Health status***
***Afousi**, 2018 (45)*	Mixed	18	54.78 ± 6.19	85.38 ± 11.23	29.38 ± 0.93	Pre-obesity	Yes	Type 2 diabetes
		17	53.12 ± 4.84	82.64 ± 11.18	29.18 ± 0.88			
***Akazawa**, 2012 (26)*	Female	11	59 ± 5	54 ± 2	22.7 ± 1.0	Normal weight	NR	Healthy individuals
***Alkatib**, 2014 (42)*	Female	8	55.2 ± 3.6	69.1 ± 15.1	NR	NR	NR	Healthy individuals
***Benda**, 2015 (27)*	Mixed	10	63 ± 8	87.6 ± 23.6	28.1 ± 7.5	Pre-obesity	Yes	Heart failure
	Male	10	64 ± 8	89.7 ± 11.9	28.9 ± 4.7			
***Boonpin**, 2017 (53)*	Female	20	55.35 ± 2.84	59.11 ± 8.9	25.22 ± 3.06	Pre-obesity	Yes	Hypertension and/or diabetes
***Borges**, 2021 (64)*	Mixed	10	57 ± 10.1	NR	23.5 ± 4	Normal weight	Yes	Chagas heart disease
***Bouaziz**, 2019 (75)*	Mixed	30	72.9 ± 2.5	77.4 ± 15.4	28.7 ± 5.6	Pre-obesity	Yes	Type 2 diabetes, hypertension, dyslipidaemia, osteoporosis, obesity
***Chacaroun**, 2020 (83)*	Mixed	12	52 ± 12	NR	31.2 ± 2.4	Obesity class 1	Yes	Obesity
		11	56 ± 11	NR	31.8 ± 3.2			
***Chasland**, 2021 (84)*	Male	21	59.7 ± 5.1	101.4 (94.7—108)	33.7 (31.7—35.5)	Obesity class 1	Yes	Healthy individuals
		20	59 ± 4.4	109.2 (102.4—116.1)	34.4 (32.4—36.4)			
***Conraads**, 2015 (85)*	Mixed	100	57 ± 8.8	84.7 ± 14.4	28.0 ± 4.4	Pre-obesity	Yes	Coronary artery disease
		100	59.9 ± 9.2	85.0 ± 13.7	28.5 ± 4.3			
***Cornelissen**, 2014 (34)*	Female	146	61.8 ± 0.73	80.4 ± 1.05	27.4 ± 0.34	Pre-obesity	Yes	Coronary artery disease
***Dall**, 2015*	Mixed	9		79.0 ± 14.3	26.7 ± 4	Pre-obesity	Yes	Heart transplant

(87)		8	51.9 ± 33-70	78.1 ± 13.3	22.94 ± 4	Normal weight		
da Silva, *2012* *(86)*	Mixed	10	50.7 ± 9.2	NR	29 ± 6	Pre-obesity	Yes	Type 2 diabetes, metabolic syndrome
		10	52.2 ± 7.5	NR	26 ± 3			
Eigendorf, *2019* *(43)*	Female	146	53 ± 4.9	72.1 ± 13.7	25.6 ± 4.4	Pre-obesity	NR	Hypertension, depression, hypothyroidism
Eleutério-Silva, *2013* *(35)*	Female	11	46.68 ± 3.43	83.18 ± 26	34.93 ± 7.54	Obesity class 1	Yes	Metabolic syndrome
Gainey, *2016* *(82)*	Mixed	11	63 ± 2	61.1 ± 3.4	26.6 ± 1.4	Pre-obesity	Yes	Type 2 diabetes
		12	58 ± 2	65.8 ± 2.7	27.01 ± 1.4			
Gelinas, *2017* *(30)*	Mixed	24	69 ± 7	NR	27.8 ± 2.8	Pre-obesity	yes	Chronic obstructive pulmonary disease
Gholami, *2020* *(46)*	Mixed	16	53.4 ± 9.1	NR	28.2 ± 2.5	Pre-obesity	Yes	Type 2 diabetes, peripheral neuropathy
Gibbs, *2012* *(47)*	Mixed	3	58.5 ± 5	NR	32.3	Obesity class 1	Yes	Type 2 diabetes
Gliemann, *2020* *(48)*	Female	10	62.3 ± 6.5	74.7 ± 14.2	NR	NR	No	Healthy individuals
Grafe, *2018* *(36)*	Mixed	15	66.1 ± 5.1	87.3 ± 12.8	27.7 ± 3.8	Pre-obesity	Yes	Coronary artery disease
Green, *2014* *(37)*	Mixed	45	41 ± 17	94.7 ± 22.3	28.6 ± 6.3	Pre-obesity	Yes	NR
		45	42 ± 15	86.0 ± 19.1	28.1 ± 5.3			
		47	42 ± 14	84.9 ± 14.4	28.0 ± 3.8			
		45	45 ± 15	80.6 ± 12.7	27.0 ± 3.9			
Greenwood, *2015* *(50)*	Mixed	13	53.9 ± 10.7	76.3 ± 15.6	26.6 ± 4.7	Pre-obesity	Yes	Renal transplant
		13	54.6 ± 10.6	79.4 ± 13.8	28.2 ± 3.6			
Greenwood, *2015* *(49)*	Mixed	16	53.8 ± 13.5	76.5 ± 15.2	27.4 ± 3.52	Pre-obesity	Yes	Chronic kidney disease
Gunnarsson, *2020* ***(51)***	Female	8	63.9 ± 5.1	65.8 ± 7.0	25.1 ± 2.9	Pre-obesity	Yes	Hypertension
		9	63.3 ± 6.9	81.1 ± 16.8	28.7 ± 6.2			
Haynes, *2021* *(52)*	Mixed	17	61.9 ± 5.4	72.8 ± 11.5	NR	NR	NR	Healthy individuals
		18	62.2 ± 7.4	77.6 ± 21.1				
Headley, *2014*	Mixed	25	58 ± 8	101.7 ± 24.9	34.9 ± 8.0	Obesity class 1	Yes	Chronic kidney disease

*(**54**)*								
Hellsten, *2012* *(**38**)*	Mixed	11	46 ± 1	76.7 ± 2.8	NR	NR	Yes	Hypertension
		10	47 ± 1	80.6 ± 7.7				
Hildreth, *2018* *(**55**)*	Male	19	66 ± 5	91.3 ± 13.0	28.9 ± 3.4	Pre-obesity	Yes	Hypertension, dyslipidemia
Hotta, *2019* *(**56**)*	Mixed	15	71 ± 2	92 ± 9.5	33 ± 3.3	Obesity class 1	Yes	Peripheral artery disease
Jaime, *2019* *(**57**)*	Female	12	64 ± 1	60.3 ± 2.3	24 ± 0.6	Normal weight	NR	Healthy individuals
Kato, *2017* *(**58**)*	Mixed	25	70 ± 11	NR	22.5 ± 3.3	Normal weight	Yes	Chronic heart failure
Keech, *2020* *(**59**)*	Mixed	29	54 ± 7	85 ± 23	27.7 ± 7	Pre-obesity	Yes	Coronary artery disease
Kim, *2019* *(**39**)*	Mixed	10	69.33 ± 2.54	62.13 ± 4.29	NR	NR	Yes	Chronic diseases or metabolic syndrome
Kirkman, *2019* *(**60**)*	Mixed	16	55 ± 13	NR	30 ± 2	Pre-obesity	Yes	Chronic kidney disease
Klonizakis, *2014* *(**61**)*	Mixed	7	64 ± 4	63 ± 13.5	NR	NR	Yes	NR
		11	64 ± 7	62.3 ± 6				
Koshiba, *2019* *(**40**)*	Female	20	60.1 ± 1.6	56.8 ± 1.9	23.4 ± 0.6	Normal weight	NR	NR
Kujawski, *2018* *(**62**)*	Mixed	27	64 ± NR	NR	26.7 ± 4	Pre-obesity	NR	Healthy individuals
		28	66 ± NR		22.94 ± 4	Normal weight		
Lee, *2019* *(**63**)*	Female	15	49.1 ± 7.9	80.9 ± 17.7	33.1 ± 7.6	Obesity class 1	Yes	Breast cancer
Lekavich, *2021* *(**65**)*	Mixed	11	53.1 ± 6.4	90.5 ± 10.4	31.7 ± 3.4	Obesity class 1	NR	Obesity, dyslipidemia
		10	47 ± 12.3	86.5 ± 12.7	29.3 ± 2.3	Pre-obesity	NR	
Luk, *2011* *(**66**)*	Mixed	32	67.7 ± 9	NR	24.7 ± 2.4	Normal weight	Yes	Healthy individuals
Magalhães, *2019* *(**67**)*	Mixed	13	58.9 ± 7.5	84.2 ± 19.2	30.2 ± 5.9	Obesity class 1	Yes	Type 2 diabetes
		16	60.4 ± 6.8	82.0 ± 13.8	31.0 ± 5.5			
Munch, *2018* *(**41**)*	Mixed	6	66 ± 2	77 ± 5	25 ± 2	Normal weight	No	Heart failure

		8	58 ± 4	89 ± 6	29.2 ± 2	Pre-obesity	Yes	
		16	60.4 ± 6.8	82.0 ± 13.8	31.0 ± 5.5	Obesity class 1	Yes	
Munch, *2018 (68)*	Mixed	14	63 ± 3	87 ± 3	28 ± 1	Pre-obesity	Yes	Heart failure
		12	59 ± 3	89 ± 5	29 ± 1			
Nishiwaki, *2015 (28)*	Male	8	45 ± 4	69.9 ± 2.2	23.6 ± 0.9	Normal weight	NR	Healthy individuals
Novaković, *2019 (69)*	Mixed	10	65.1 ± 7.6	NR	30.4 ± 6.2	Obesity class 1	Yes	Peripheral artery disease
		11	65.6 ± 11	NR	27.3 ± 3.3	Pre-obesity		
Nualnim, *2012 (70)*	Mixed	24	58 ± 2	81 ± 3	29 ± 1	Pre-obesity	No	Healthy individuals
Nyberg, *2016 (29)*	Female	20	50 ± 0	69.2 ± 1.4	24.4 ± 0.5	Normal weight	NR	Healthy individuals
		16	54 ± 1	64.4 ± 1.8	23.1 ± 0.5			
O'Brien, *2020 (71)*	Mixed	12	68 ± 5	NR	25.9 ± 3.1	Pre-obesity	Yes	Hypertension, asthmatic, hypothyroidism
		12	68 ± 6	NR	25.2 ± 3.6			
		14	66 ± 7	NR	27.2 ± 5.1			
Park SY, *2020 (72)*	Mixed	28	60 ± 9	55 ± 7.4	22.8 ± 2.8	Normal weight	Yes	NR
		25	60 ± 10	56.5 ± 5.7	22.5 ± 2.4			
Park S-Y, *2019 (73)*	Mixed	42	70 ± 10	54.1 ± 7.1	20.4 ± 2.1	Normal weight	Yes	Peripheral artery disease
Park W, *2020 (74)*	Male	10	69.1 ± 0.9	70.7 ± 3.8	26.2 ± 0.5	Pre-obesity	Yes	Peripheral artery disease
Pierce, *2011 (30)*	Mixed	26	63 ± 1	73.5 ± 2.9	25.3 ± 0.7	Pre-obesity	Yes	Healthy individuals
Pierce, *2016 (31)*	Mixed	14	63 ± 1	71 ± 2.6	23.5 ± 0.6	Normal weight	Yes	NR
Schmidt, *2014 (76)*	Male	9	68 ± 4	77.7 ± 9.4	26.1 ± 3.9	Pre-obesity	NR	Healthy individuals
		9	69.1 ± 3.1	85.8 ± 12	27.4 ± 2.8			
Schreuder, *2014 (77)*	Mixed	10	57 ± 6	106 ± 18	30.9 ± 4.1	Obesity class 1	Yes	Type 2 diabetes
		9	52 ± 8	115 ± 23	36.0 ± 6.5	Obesity class 2		
Shinno, *2017 (78)*	Female	11	47.9 ± 2.2	54 ± 11.6	NR	NR	No	Healthy individuals
		11	46.9 ± 3.2	53.6 ± 8.6				
Tanahashi, *2017 (32)*	Mixed	26	61 ± 7	58.7±7.3	23.1±2.4	Normal weight	NR	Healthy individuals

van Zanten, *2019* *(**33**)*	Mixed	50	50 ± 10	NR	28.8 ± 1.77	Pre-obesity	Yes	Rheumatoid arthritis
Vinet, *2018* *(**79**)*	Mixed	17	58.2 ± 3.8	92 ± 13.6	32.8 ± 0.7	Obesity class 1	Yes	Metabolic syndrome
		21	67.7 ± 4.2	85.2 ± 13.1	32.3 ± 13.1			
		24	59.2 ± 5.1	89.4 ± 12	33.7 ± 3.6			
Williams, *2013* *(**80**)*	Mixed	25	66.9 ± 4.3	78.5 ± 15.6	28.1 ± 4.4	Pre-obesity	Yes	Healthy individuals
		24	66.4 ± 4.3	78.2 ± 15.2	28.0 ± 4.9			
Wong, *2014* *(**81**)*	Female	15	57 ± 1	87.6 ± 4.1	34.2 ± 1.2	Obesity class 1	NR	Healthy individuals

* Nutritional status classifications accordingly National Institute of Health (*NIH*) and the World Health Organization (*WHO*) for White, Hispanic, and Black individuals (**1**). Body mass index (***BMI***); Not reported (***NR***). **1.** Weir CB, Jan A. BMI classification percentile and cut-off points. 2019;
