# Supplementary material for: Impact of Exercise on Vascular Function in Middle-Aged and Older Adults: A Scoping Review
Source: Sports (Basel). 2022 Dec 14;10(12):208. doi: 10.3390/sports10120208 (PMC9786288; doi:10.3390/sports10120208)
Supplement: Supplementary file 1 [file sports-10-00208-s001.zip › Table S3- Vascular outcome.pdf]

Table S3. Vascular outcomes

**Macrovascular Outcomes**		
	PWV (n = 21)	(Greenwood, Koufaki, Mercer, Rush, et al. 2015) [63](Gainey et al. 2016) [92](Bouaziz et al. 2019) [50] (Kirkman et al. 2019) [72](Greenwood, Koufaki, Mercer, MacLaughlin, et al. 2015)[62] (Nualnim et al. 2012)[81] (Pierce et al. 2016)[44](Novaković et al. 2019) [80](Headley et al. 2014)[66] (Park, Kwak, and Pekas 2019) [84](Keech et al. 2020)[71] (Chacaroun et al. 2020)[51] (Magalhães et al. 2019)[78] (Wong and Figueroa 2014) [91]](Kujawski et al. 2018)[74] (Koshiba and Maeshima 2019)[39] (S.-Y. Park et al. 2020) [84] (Eigendorf et al. 2019)[56] (Jaime et al. 2019)[69] (Boonpim et al. 2017)[48] (W. Park et al. 2020)[85]
	ALX (n = 12)	(Schmidt et al. 2014)[86] (Kirkman et al. 2019) [72](Greenwood, Koufaki, Mercer, MacLaughlin, et al. 2015)[62] (Nualnim et al. 2012) [81](Pierce et al. 2016)[44] (Headley et al. 2014)[66] (Keech et al. 2020)[71] (Wong and Figueroa 2014)[91] (Jaime et al. 2019)[69] (Williams et al. 2013)[90] (Dall et al. 2015)[55] (Eleuterio-Silva et al. 2013)[34] (Schmidt et al. 2014)[86]
	FMD (n = 29)	(Vinet et al. 2018)[89] (Veldhuijzen van Zanten et al. 2019)[46] (Gholami, Nazari, and Alimi 2020) [59](Luk et al. 2012)[79] (Gibbs et al. 2012)[60] (Lee et al. 2019)[75] (Conraads et al. 2015)[53] (Gainey et al. 2016)[92] (Bouaziz et al. 2019)[50] (Haynes et al. 2021)[63] (Chasland et al. 2021)[52] (Ghardashi Afousi et al. 2018)[58] (Hildreth et al. 2018)[67[(Nualnim et al. 2012)[81] (Klonizakis et al. 2014)[73] (Novaković et al. 2019) [80] (Green et al. 2014) [36](Schreuder et al. 2014)[87] (Gelinas et al. 2017)[57](Hotta et al. 2019)[68] (Jaime et al. 2019) [69](O'Brien et al. 2020)[82[(Benda et al. 2015)[32](Gregers Winding Munch et al. 2018) [40](Kirkman et al. 2019)[72] (Akazawa et al. 2012)[31] (Grafe et al. 2018)[35] (Pierce et al. 2011)[43] (Cornelissen et al. 2014) [33]
	GTN (n = 11)	(Veldhuijzen van Zanten et al. 2019)[46] (Haynes et al. 2021)[65] (Chasland et al. 2021) [52](Klonizakis et al. 2014)[73] (Green et al. 2014)[36] (Schreuder et al. 2014) [87] (Benda et al. 2015)[32] (da Silva et al. 2012)[54] (Luk et al. 2012) [77]Gibbs et al. 2012)[60] (Vinet et al. 2018)[89
	ABI (n = 5)	(Gainey et al. 2016)[92] (Novaković et al. 2019)[80] (Kim and Kim 2019)[38] (Park et al. 2019) [84](S.-Y. Park et al. 2020) [83]
	CAVI (n = 3)	(Nishiwaki et al. 2015) [41] (Kim and Kim 2019)[38] (Koshiba and Maeshima 2019) [39]
	PSR (n = 1)	(Lekavich et al. 2021) [76]
	IMT (n = 6)	(Gholami et al. 2020) [59] (Lee et al. 2019) [75] (Hildreth et al. 2018) [67] (Gelinas et al. 2017) [57] (Magalhães et al. 2019) [78] (Tanahashi et al. 2017) [45]
**Microvascular Outcomes**		
	ACh (n = 9)	(Borges et al. 2021) [49] (Veldhuijzen van Zanten et al. 2019) [46] (Gregers W Munch et al. 2018) [40] (Vinet et al. 2018) [89] (Alkhatib and Klonizakis 2014) [47] (Gunnarsson et al. 2020) [64] (Nyberg et al. 2016) [42] (Hellsten et al. 2012) [37] (Gliemann et al. 2020) [61]
	SNP (n = 7)	(Borges et al. 2021) [49] (Veldhuijzen van Zanten et al. 2019) [46] (Kirkman et al. 2019) [72] (Gregers W Munch et al. 2018) [40] (Vinet et al. 2018) [89] (Alkhatib and Klonizakis 2014) [47] (Gunnarsson et al. 2020) [64]
	RH / RHI (n = 12)	(Kirkman et al. 2019) [72] (Dall et al. 2015) [34] (Gregers Winding Munch et al. 2018) [40] (Grafe et al. 2018) [43] (da Silva et al. 2012) [54] (Borges et al. 2021) [49] (Chacaroun et al. 2020) [51] (Klonizakis et al. 2014) [73] (Pierce et al. 2011) [43] (Cornelissen et al. 2014) [33] (Kato et al. 2017) [70] (Shinno et al. 2017) [88] (Schmidt et al. 2014) [86]

Ankle brachial index (**ABI**); Acetylcholine (**ACh**)-mediated vasodilation; Augmentation index (**ALX**); Cardio-ankle vascular index (**CAVI**); Flow-mediated dilatation (**FMD**); Glyceryl trinitrate-induced dilation (**GTN**); Intima–media

thickness (**IMT**); Nitroglycerine mediated dilatation (**NMD**); Pulse stiffening ratio (**PSR**); Pulse wave velocity (**PWV**); Reactive hyperemia (**RH**); Reactive hyperemia/Reactive hyperemia index (**RH/RHI**), Sodium nitroprusside (**SNP**) endothelium-independent relaxant.
